# Blebbisomes as autonomous communication hubs

**DOI:** 10.20517/evcna.2025.38

**Published:** 2025-07-11

**Authors:** Ying Gao, Tiebang Kang

**Affiliations:** State Key Laboratory of Oncology in South China, Guangdong Provincial Clinical Research Center for Cancer, Sun Yat-sen University Cancer Center, Guangzhou 510060, Guangdong, China.

**Keywords:** Blebbisomes, EVs, communication

## Abstract

Blebbisomes represent a newly identified class of large extracellular vesicles (EVs) that exhibit unique functional capabilities in intercellular communication. Beyond their motility, blebbisomes engage in complex vesicle trafficking functions. Strikingly, they are capable of both internalizing and secreting other EVs, essentially acting as intermediaries or “hubs” in intercellular communication networks.

## MAIN TEXT

Cells communicate via extracellular vesicles (EVs)-membrane-bound particles (30 nm to several µm) that shuttle proteins, lipids, and nucleic acids to target cells, orchestrating physiological and pathological processes. While exosomes (30-120 nm, endosomal origin) and microvesicles (150-1,000 nm, plasma membrane-derived) are well-characterized, recent discoveries of exophers, migrasomes, retractosomes, oncosomes, mitopher, Rafeesome-R-EV, and other EVs underscore the incompleteness of our understanding of EV diversity^[1-8]^. This knowledge gap has paved the way for identifying novel EV classes that redefine intercellular communication.

**Figure 1:**
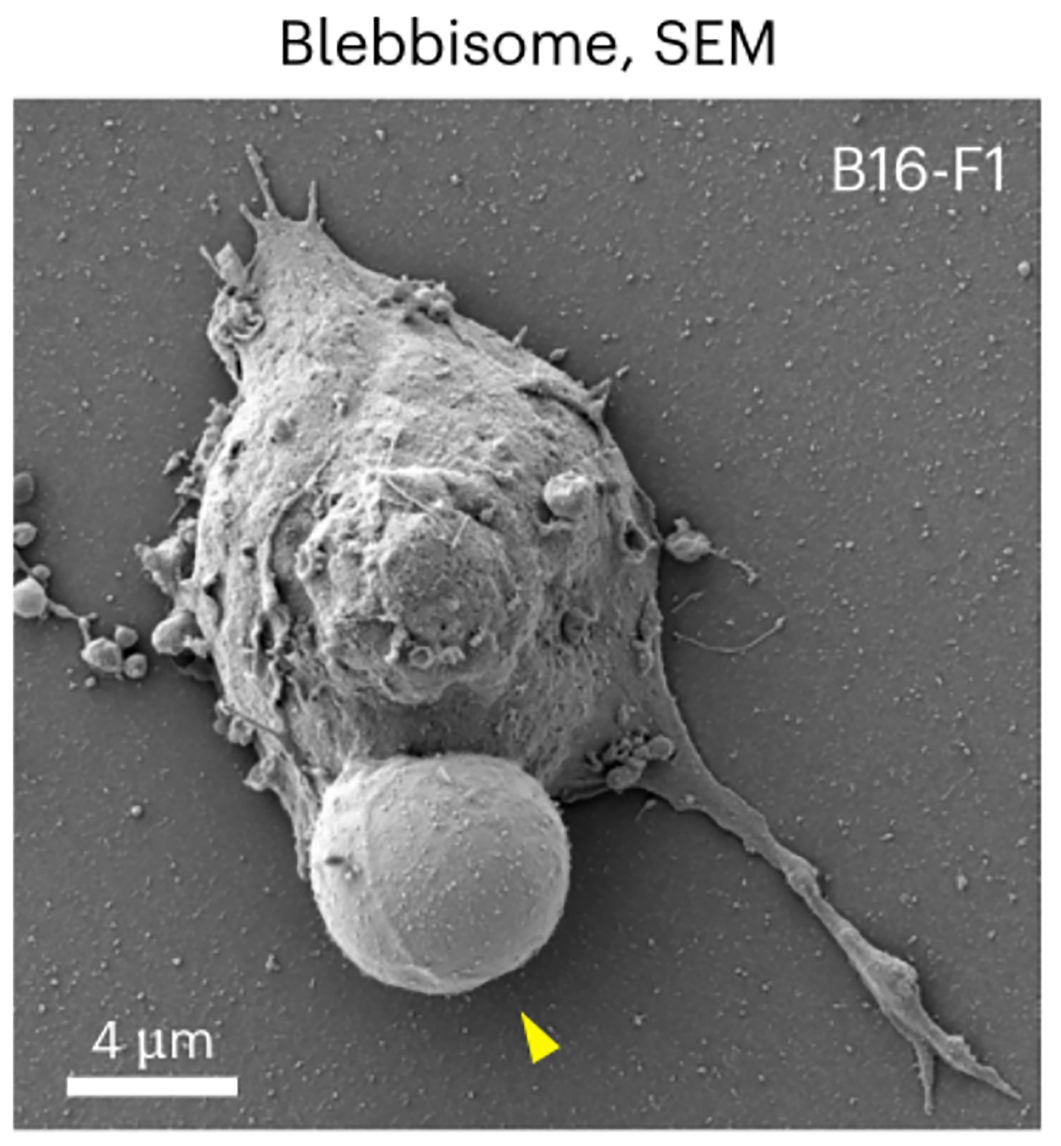
An SEM micrograph of B16-F1 blebbisome displaying a large characteristic bleb (arrowhead)^[9]^. Adapted from Ref.^[9]^(Open Access). SEM: Scanning electron microscopy.

A groundbreaking study in *Nature Cell Biology* reports the discovery of an entirely new class of EVs called blebbisomes - exceptionally large, cell-like vesicles shed by cells [Figure 1]^[9]^. Blebbisomes are striking in size, with an average diameter of ~10 µm and reaching up to 20 µm, making them the largest EVs documented to date. They were first observed through live-cell imaging as balloon-like membrane blebs that, upon detachment, persisted as free vesicles exhibiting dynamic “blebbing” of their own membrane. This pronounced blebbing activity - continual expansion and retraction of their membrane - inspired the name blebbisome. Once released, blebbisomes remain motile and independent, moving in the extracellular environment without any tether to their parent cell. Intriguingly, these massive vesicles were found to be produced not only by cancer cell lines but also by normal cells, indicating that blebbisome formation is a general cellular phenomenon rather than an artifact of transformed cells. The presence of blebbisomes has been confirmed across human and murine cells, underscoring their broad relevance in biology.

Blebbisomes display remarkable ultrastructural organization, encapsulating a comprehensive array of functional organelles within a nucleus-free cytoplasmic compartment. They effectively package a microcosm of the parent cell's cytoplasm - containing intact mitochondria, endoplasmic reticulum (ER), and other organelles, while excluding nuclear material. This unique composition enables autonomous metabolic activity (supported by mitochondria maintaining mitochondrial membrane potential and ATP synthesis) without replicative capacity. Crucially, blebbisomes harbor fully functional organelles rather than the damaged components typically sequestered in other EVs for disposal. This preservation of organelle integrity, particularly of “healthy” polarized mitochondria, distinguishes blebbisomes from conventional EVs and suggests active maintenance of their internal environment. Their extracellular motility, potentially driven by both intrinsic blebbing dynamics and environmental interactions, further reinforces their cell-like autonomy within the extracellular milieu.

Blebbisomes join a growing family of large extracellular vesicles (lEVs) (> 1 µm) that have come to light in recent years, including migrasomes, exophers, and large oncosomes. Despite some superficial similarity in size, blebbisomes are distinct from migrasomes, exophers, and oncosomes in several key ways. Blebbisomes are considerably larger - often double the diameter of migrasomes or exophers, and exceeding even the upper size of oncosomes. Unlike migrasomes, which are numerous and small in each retraction, blebbisomes emerge one at a time, as a single giant vesicle per bleb retraction. In terms of content, blebbisomes carry intact functional organelles (notably healthy mitochondria), whereas exophers and migrasomes tend to sequester damaged mitochondria and debris destined for removal^[3,10]^. Proteomic comparisons indicate blebbisomes are rich in ER proteins and endosomal markers, whereas large oncosomes contain little ER and their mitochondrial proteins likely represent remnants of dysfunctional organelles. The distinct biogenesis (myosin-driven bleb scission *vs*. migrasome fiber budding or oncosome membrane shedding) and the presence of a full suite of organelles set blebbisomes apart as a novel category of EV. They are essentially one bleb away from being a cell-a description that encapsulates their near-cellular complexity relative to other EVs.

Beyond their motile capacity, blebbisomes exhibit sophisticated bidirectional vesicle trafficking capabilities that establish them as nodal points in intercellular communication networks. These giant vesicles demonstrate the remarkable ability to both internalize and secrete smaller EVs, including exosomes and microvesicles. Experimental evidence reveals that isolated blebbisomes actively engulf surrounding EVs through an endocytic process, supported by the presence of multivesicular endosomes (MVEs) and endocytic compartments within their cytoplasm. Notably, purified blebbisomes generate and release nano-vesicles expressing characteristic exosomal markers (syntenin-1, TSG101) and microvesicle markers (annexins A1/A2), indicating preservation of the endosomal sorting complex required for transport (ESCRT) machinery. This bidirectional trafficking capacity enables blebbisomes to function as cellular microprocessors - receiving, integrating, and redistributing molecular information within tissues.

The presence of functional mitochondria enables blebbisomes to sustain these activities for extended periods (up to 72 h *in vitro*), significantly exceeding the lifespan of conventional EVs. This exceptional longevity, combined with their capacity for both signal reception (via EV internalization and surface receptor engagement) and signal transmission (through vesicle secretion and immune checkpoint presentation), establishes blebbisomes as persistent, motile signaling platforms. The discovery of blebbisomes fundamentally challenges the conventional paradigm of EVs as passive cargo carriers, instead revealing a spectrum of extracellular organelles with varying degrees of cellular autonomy. Their capacity to actively participate in signal processing and redistribution suggests an unprecedented level of sophistication in intercellular communication networks. This challenges the traditional view of EVs as inert packages and suggests that some EVs are dynamic participants in cell-to-cell crosstalk.

One of the most provocative discoveries about blebbisomes is their enrichment in immune inhibitory proteins, hinting at a role in modulating immune responses. Proteomic analysis of cancer-cell-derived blebbisomes revealed a plethora of immune checkpoint and “don’t-eat-me” ligands on their surface. These include well-known checkpoint molecules such as PD-L1 and PD-L2 (which engage PD-1 receptors on T cells), B7-H3 and VISTA (which can suppress T cell activation), HLA-E (which interacts with inhibitory NKG2A receptors on natural killer cells), as well as CD73 and CD47 among others. Notably, many of these factors were present at higher levels on blebbisomes than on smaller EVs from the same cells. This extensive display of inhibitory ligands suggests that blebbisomes could serve as potent agents of immune suppression in the tumor microenvironment. By ferrying multiple checkpoint ligands, a single blebbisome might interact with diverse immune cell types and shut down antitumor immunity on multiple fronts. This raises the possibility that tumors shedding blebbisomes gain a survival advantage, using these large EVs as decoys or suppressive messengers to create an immune-privileged niche. The authors suggest that the role of blebbisomes in immunosuppression and immune evasion warrants further investigation. Targeting blebbisomes or their cargo could emerge as a novel therapeutic angle to enhance antitumor immunity if their immunomodulatory functions are confirmed *in vivo*.

In conclusion, the identification of blebbisomes expands the landscape of extracellular vesicle biology. These massive, organelle-rich vesicles stand at the boundary between cells and vesicles, possessing many attributes of living cells except an autonomous replicative capacity. Their existence opens up exciting questions: What triggers a cell to form a blebbisome versus other EVs? What are the markers of blebbisome? How do blebbisomes interact with recipient cells or other EVs in a tissue? And can we exploit blebbisomes for therapeutic benefit - perhaps as natural delivery vehicles or targets to disrupt tumor communication? As researchers begin to explore these questions, blebbisomes are poised to yield fresh insights into fundamental mechanisms of intercellular communication and offer potential avenues for innovation in diagnostics and therapy. The discovery of blebbisomes thus highlights how much remains to be learned about the secret life of cells beyond their boundaries.

## DECLARATIONS

### Copyright

© The Author(s) 2025.
